# Renal Denervation: A Potential Novel Treatment for Type 2 Diabetes Mellitus?

**DOI:** 10.1097/MD.0000000000001932

**Published:** 2015-11-06

**Authors:** Tao Pan, Jin-he Guo, Gao-jun Teng

**Affiliations:** From the Department of Interventional and Vascular Surgery, Zhongda Hospital, Southeast University, Jiangsu, China.

## Abstract

Type 2 diabetes mellitus (T2DM) is a group of metabolic diseases of multiple etiologies. Although great progress has been made, researchers are still working on the pathogenesis of T2DM and how to best use the treatments available. Aside from several novel pharmacological approaches, catheter-based sympathetic renal denervation (RDN) has gained a significant role in resistant hypertension, as well as improvements in glycemic control in T2DM.

In this article, we will summarize herein the role sympathetic activation plays in the progression of T2DM and review the recent clinical RDN experience in glucose metabolism.

We performed systematic review in online databases, including PubMed, EmBase, and Web of Science, from inception until 2015.

Studies were included if a statistical relationship was investigated between RDN and T2DM.

The quality of each included study was assessed by Newcastle–Ottawa scale score. To synthesize these studies, a random-effects model or a fixed-effects model was applied as appropriate. Then, we calculated heterogeneity, performed sensitivity analysis, tested publication bias, and did meta-regression analysis. Finally, we identified 4 eligible articles.

In most studies, RDN achieved via novel catheter-based approach using radiofrequency energy has gained a significant role in resistant hypertension, as well as improvements in glycemic control in T2DM. But the DREAMS-Study showed that RDN did not change median insulin sensitivity nor systemic sympathetic activity.

Firstly, the current published studies lacked a proper control group, along with the sample capacity was small. Also, data obtained in the subgroups of diabetic patients were not separately analyzed and the follow-up period was very short. In addition, a reduction in blood pressure accounts for the improvements in glucose metabolism and insulin resistance cannot be excluded.

If the favorable result of better glucose metabolism is confirmed in large-scale, randomized studies, RDN may emerge as a novel therapeutic option for patients with T2DM.

## INTRODUCTION

Diabetes mellitus (DM) is a group of metabolic diseases that resulted from alterations in insulin secretion or insulin action, which is characterized by impairment in glucose, protein, and lipid metabolism.^1^ Among the 4 types of DM, type 2 DM (T2DM) is the most prevalent and makes up 90% to 95% of all the diabetic patients,^2,3^ whose pathophysiology includes insulin resistance, hyperglycemia, and a variable degree of insulin secretory deficiency.^4^ Patients with T2DM are at a high risk of macrovascular events, doubling the risk of developing acute myocardial infarction and stroke,^5^ indicating a higher mortality rate in patients affected by heart failure alongside T2DM than nondiabetic ones^6^ and leading to about 8 years shorter of life expectance than the general population.^7^ Importantly, unless urgent measures are taken, 1 in 19 adults will be affected by DM by 2030.^8^

Over 3 decades ago, Asmal and Marble^9^ stated that “despite the availability of oral hypoglycaemic drugs for nearly 30 years, their precise mode of action and role in the management of diabetes mellitus remains poorly defined and controversial.” Until now, although great progress has been made, researchers are still working on the pathogenesis of T2DM and how to best use the treatments available.^10^ The past few years have witnessed the international interests of clinicians and researchers on the approach of renal denervation (RDN) by catheter using radiofrequency energy as a novel treatment for resistant hypertension.^11^ In some RDN researches, aside from better blood pressure control, a fall of fasting glucose and an increase in insulin sensitivity had been observed.^12–14^ This improvement in glycemic control likely resulted from attenuation in sympathetic nervous system activity and is accompanied with a reduction in insulin resistance. However, very recent findings of the DREAMS-Study^15^ showed that fasting glucose and median insulin sensitivity did not change after RDN and no effect in systemic sympathetic activity was observed. Therefore, further evaluation in rigorously designed clinical trials will be necessary before any definite conclusion can be drawn.

In this article, we will summarize herein the role sympathetic activation plays in the progression of T2DM and review the recent clinical RDN experience in glucose metabolism.

### Sympathetic Nervous System and T2DM

Awareness of the role of the sympathetic nervous system in T2DM has captured scientific attention with the possibility of catheter-based RDN as a novel therapeutic option.

### Elevated Sympathetic Nerve Activity in T2DM

A great many clinical and epidemiological investigations have observed elevated sympathetic nerve activity in T2DM.^16–20^ Huggett et al^18^ examined muscle sympathetic nerve activity (MSNA), which has been shown to correlate well with sensory, somatomotor, and autonomic effector responses of sympathetic activity,^21^ in 4 groups of subjects, patients with T2DM alone, essential hypertension alone, essential hypertension accompanied with T2DM, and healthy controls. They found highest MSNA in hypertensive-T2DM patients, as well as higher MSNA in essential hypertension alone and T2DM alone patients comparing with matched subjects. Fasting insulin levels were greater in hypertensive-T2DM and T2DM alone patients than in hypertensive patients and healthy controls. Their observations provided the proof that T2DM patients had a marked increase in sympathetic nervous system activity, even in the presence of normal blood pressure. In support of this contention, their subsequent study^19^ demonstrated that patients with T2DM had both greater resting MSNA and single-unit MSNA, although with similar reflex responses, baroreflex sensitivity, and sympathovagal balance controlling the heart period. Furthermore, very recent findings showed that T2DM patients had higher resting MSNA burst incidence, increased arterial norepinephrine levels, lower plasma norepinephrine clearance, and reduced neuronal reuptake compared with controls; besides, norepinephrine spillover responses to glucose ingestion were blunted in subjects with T2DM.^20^ Moreover, in another investigation conducted by Huggett et al,^22^ elevated sympathetic nerve activity also occurred in normal nondiabetic offspring of patients with T2DM in proportion to their plasma insulin levels and resistance, indicating the presence of a mechanistic link between sympathetic activation and hyperinsulinemia. More recently, Santulli et al^23,24^ stated that systemically circulating or locally released sympathetic catecholamines may activate α1 and β2 adrenergic receptors, which provides fine-tuning to the endocrine pancreas activity and insulin secretion. These observations suggest that the overactivity of sympathetic nervous system may be strongly associated with T2DM.

### The Potential Mechanisms Linking Sympathetic Activation to T2DM

The task now becomes to demonstrate how the sympathetic nervous system may result in T2DM. Since 1664, Thomas Willis first described the sympathetic nervous system in detail^25^; researches had been carried on the role of it. Besides in cardiovascular regulation, the activation of sympathetic nervous system also has profound metabolic effects leading to increased levels of fatty acids in plasma, increased hepatic gluconeogenesis, and alterations in pancreatic insulin release.^26,27^ Sympathetic activation could contribute to the development of insulin resistance,^28^ which cells fail to respond to insulin and is generally considered as a key feature in the pathogenesis of T2DM.^29^ In a study of isolated perfused forearm models in 14 healthy volunteers, an increase was found in sympathetic nervous system activation, which can cause acute insulin resistance in the forearm.^30^ Moreover, the subsequent study in a cohort of 433 young, nonobese, normotensive men^31^ showed that baseline plasma noradrenaline levels, which can provide information on sympathetic activity,^32^ independently predicted a rise in hyperinsulinemia, body mass index (BMI), and blood pressure in 5 years. Similarly, it has been demonstrated that in response to a cold pressor test, which predominantly activates alpha adrenergic responses, noradrenaline independently predicted insulin resistance at 18-year follow-up.^33^ These findings were supported by observations that acute activation of endogenous noradrenaline release antagonized insulin-mediated glucose uptake in forearm skeletal muscle.^34^ According to Jamerson et al,^30^ a decrease in blood flow after the raise of noradrenaline concentration could impair the ability of cells to transport glucose through their membranes, which is probably the result of alpha-adrenoceptor mediated vasoconstriction caused by sympathetic overactivity. This physiological activity could reduce the number of open capillaries and increase the distance, which insulin has to travel from the intravascular compartment to reach the cell membrane.^35^ Collectively, these studies suggest that pathological activation of sympathetic nervous system plays a significant role in the process of insulin resistance, making patients more prone to the development of impaired glucose tolerance and T2DM.^18,35^

On the contrary, almost 30 years ago, Landsberg^36^ proposed a link between insulin resistance and activation of the sympathetic nervous system. Thereafter, insulin resistance could lead to an increase in sympathetic nervous system activation has been confirmed.^37^ Subsequent studies in healthy volunteers supported that infusions of insulin increased MSNA, which appeared to be independent of the vasodilatory effects of insulin.^38^ Then, it comes to a “chicken and egg” scenario; in other words, which abnormality comes first and originates the vicious circle that maintains both sympathetic overactivity and insulin resistance.^35^ Masuo et al^28^ found that in the development of hypertension, Japanese individuals showed an increase in both plasma noradrenaline and insulin levels, while 10 years earlier they only showed an increase in plasma noradrenaline. This suggested that sympathetic activation possibly causes insulin resistance in some conditions. It is vital to know whether this is also the case for T2DM to implement the most effective therapeutic measures.

Above all, there is the abundant evidence for an important role of activation of the sympathetic nervous system in insulin resistance, which is considered essential in the pathogenesis of T2DM.

### Sympathoinhibition may Improve Glycemic Control

An experiment of cats, which underwent total removal of the sympathetic nervous chain from the stellate to the pelvic ganglia, showed that normal cats experienced an average increase in blood glucose of 34.1% compared with an average decrease of 0.02% in the sympathectomized cats.^39^ Given its unfavorable major side effects profile and the high mortality rate, this approach had not been used in clinical. Lately, studies showed that sympathoinhibition would be expected to improve glycemic control.^40^ Therefore, it appears possible to propose a novel procedure that abolishes efferent and afferent renal nerve activity may be helpful to improve plasma glucose concentrations.

### Clinical Studies on RDN

Recently, a novel catheter-based approach selectively targeting the renal sympathetic nerves using radiofrequency ablation technology has been developed. It was first assessed in Symplicity HTN-1 trial,^41^ and a sustained decrease of blood pressure at 3-year follow-up was observed.^42^ MSNA were also decreased after RDN,^43^ indicating a reduction in overall sympathetic tone. The following multicenter, randomized Symplicity HTN-2 trial showed that RDN was more effective at blood pressure reduction than medical therapy alone.^44,45^ However, the findings of the Symplicity HTN-3 trial,^46^ a prospective, single-blind, randomized, sham-controlled trial, contradicted those of the Symplicity HTN-1 and HTN-2 trials. In that trial, the reduction of blood pressure after RDN was not significant as compared with a sham procedure.^46^ Therefore, more clinical trials are needed before any definite conclusion can be drawn.

Besides resistant hypertension, RDN may also benefit other diseases characterized by overactivity of sympathetic nervous system. The EnligHTN I trial^47^ showed that RDN significantly reduced average heart rate; besides, patients with heart failure may benefit from this treatment.^48^ Moreover, a reduction in left ventricular mass and an improvement of diastolic parameters were reported after RDN^49^; atrial and ventricular arrhythmias have shown to be improved as well.^50^ In addition, RDN improved renal resistance indices and decreased the severity of macroalbuminuria and microalbuminuria even in the absence of an improvement in the glomerular filtration rate (GFR).^51^ Interestingly, RDN improved glucose metabolism and insulin sensitivity in hypertensive patients with T2DM,^12–14^ suggesting that this approach might help with glycemic control in addition to hypertension control.

### Effects of RDN on Glucose Metabolism

In 1998, Huang et al^52^ performed experiments in male Sprague-Dawley rats, making hyperinsulinemic by insulin infusion via osmotic minipumps implanted subcutaneously, to evaluate the role of the renal nerves. They used surgically stripping all visible renal nerves along renal arteries and veins to obtain bilateral RDN, which depleted renal norepinephrine stores and prevented the development of hyperinsulinemia-induced hypertension. After hypertension model had been fully established, bilateral RDN reversed the elevated systolic blood pressure to normal within 2 weeks,^52^ suggesting that RDN could not only prevent hypertension but also reverse the pressor effects of insulin. In addition, the possible confounding role of insulin-induced glucose alterations was shut out by using the euglycemic clamp approach to keep constant blood glucose levels.^53^

On the basis of this background, some clinicians have experimented catheter-based RDN on glycemic control, and these studies (Table 1) were based on the theory that sympathetic overactivity can induce insulin resistance and hyperinsulinemia.^28^ Mahfoud et al^12^ investigated the effect of RDN on glucose metabolism and insulin resistance in patients with resistant hypertension. In their study, 50 patients with resistant hypertension were divided into RDN group (N = 37) and conventional treatment group (N = 13), of whom 20 (40%) were T2DM under metformin, glicazide, or combined therapy, while none was under insulin treatment. Three months after treatment, besides mean blood pressure reduction by 32 and 12 mm Hg for systolic and diastolic, respectively, a significant improvement in glucose metabolism was found in the RDN group. Specifically, fasting glucose was reduced from 118 ± 3.4 to 108 ± 3.8 mg/dL (*P* = 0.039), insulin levels from 20.8 ± 3.0 to 9.3 ± 2.5 μIU/mL (*P* = 0.006), C-peptide levels decreased from 5.3 ± 0.6 to 3.0 ± 0.9 ng/mL (*P* = 0.002), and homeostasis model assessment–insulin resistance (HOMA-IR) improved from 6.0 ± 0.9 to 2.4 ± 0.8 (*P* = 0.001) significantly. In addition, mean 2-hour glucose levels significantly reduced by 27 mg/dL (*P* = 0.012) following oral glucose tolerance test, although there were no changes in blood pressure or metabolic markers in the control group. Meanwhile, BMI and antihypertensive treatment remained unchanged during this period.^12^ However, the glycosylated hemoglobin A1c (HbA1c) levels remained nearly at baseline values and did not change significantly during 3-month follow-up. As the level of HbA1c correlates with the risk of long-term diabetes complications,^54^ it still needs to be determined that whether this may be due to short-term life span of red blood cells or other factors. This was the first study showing that RDN could reduce insulin resistance and improve glycemic control, which might by reducing sympathetic drive. The possibility observed in this research may open a promising nonpharmacological strategy for T2DM patients.

**TABLE 1:**
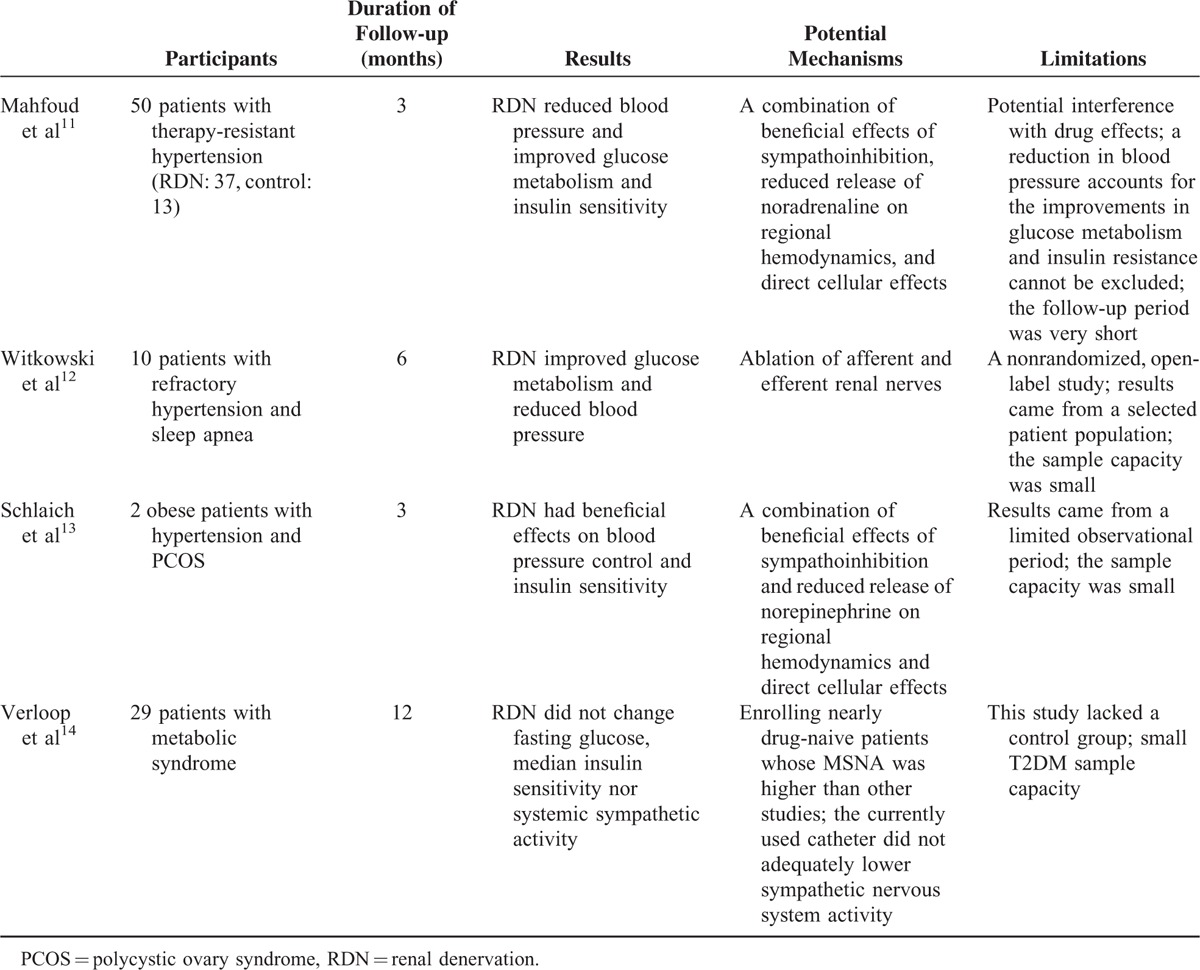
Studies of Catheter-Based RDN on Glycemic Control

The favorable effect of RDN on glucose metabolism is supported by investigations of 2 female patients with polycystic ovary syndrome (PCOS),^14^ a disease characterized by insulin resistance, obesity, diabetes, and blood pressure elevation related to sympathetic nervous activation.^55^ After both patients took RDN, fasting plasma glucose declined and insulin sensitivity improved by 17.5% in the presence of unaltered bodyweight at 3-month follow-up. Moreover, the improvements in glucose metabolism, reduction in sympathetic activity measured by MSNA, and whole-body norepinephrine spillover was observed.^14^ In addition, glomerular hyperfiltration and urinary albumin excretion were also reduced, indicating that RDN might serve as a therapeutic intervention to attenuate the progression of renal injury in diabetic nephropathy, which had been proved in an animal model.^56^ In that study, renal norepinephrine content was significantly raised following diabetic induction in streptozotocin-induced DM in (mRen-2)27 rats and ablated after denervation. Immunohistochemistry and protein densitometric analysis of diabetic-innervated kidneys revealed that RDN significantly reduced protein expression of fibrotic markers, attenuated albuminuria, and prevented the loss of glomerular podocin expression,^56^ indicating the potential of RDN to slow the progression of diabetes-induced nephropathy. Nonetheless, the available data are only from experimental models; whether RDN has similar effects in T2DM patients and could help to prevent the development of diabetes remains to be explored.

Another potential beneficial effect of RDN on glucose metabolism came from study carried out in 10 obese patients with resistant hypertension and obstructive sleep apnea syndrome (OSAS),^13^ which is characterized by an increased sympathetic activity.^57^ Six months after RDN, aside from significant reduction in blood pressure (-34/-13 mm Hg; *P* < 0.01) and apnea–hypopnea index (16.3 vs 4.5 events per hour; *P* = 0.059), plasma glucose concentration 2-hour after oral glucose tolerance test significantly reduced from 7.0 to 6.4 mmol/L (*P* = 0.05), and HbA1c decreased from 6.1% to 5.6% (*P* < 0.05).^13^ However, alterations in fasting plasma glucose have not been found, possibly because of small number of diabetic patients in this study. The mechanisms by which RDN affects insulin metabolism remain unknown. The possibilities may be complex, such as a decrease in central sympathetic outflow indicating an alteration in afferent sympathetic drive, an inhibition of the renin-angiotensin-aldosterone-system, a decrease in vascular alpha adrenergic tone, resulting in skeletal muscle vasodilation, an increased sensitivity to the nonesterified fatty acid lowering effects of insulin, and an improved glucose transport to skeletal muscle.^53,58,59^

Nevertheless, the DREAMS-Study,^15^ investigating the effects of RDN on insulin sensitivity and blood pressure in patients with metabolic syndrome, contradicted the result of better glucose metabolism of those researches mentioned above.^12–14^ In that study, 29 patients fulfilling the criteria for metabolic syndrome were enrolled, of whom 5 (17%) were T2DM. MSNA and heart rate variability (HRV) measurements were performed to assess sympathetic activity. Fasting glucose changed from 7.2 ± 1.7 to 7.4 ± 2.6 mmol/L at 6-month follow-up (*P* = 0.34) and 7.0 ± 1.3 mmol/L at 12-month follow-up (*P* = 0.34). Median insulin sensitivity, as assessed by the Simple Index assessing Insulin Sensitivity oral glucose tolerance test (SIiSOGTT), did not change at half- and 1-year follow-up (*P* = 0.60 and *P* = 0.77, respectively). Although decreased numerically by −0.55 ± 3.7 (*P* = 0.43) 1 year after RDN, HOMA-IR did not reach any statistical significance at 6- and 12-month follow-up. One year after treatment, mean ambulatory 24-hour blood pressure was reduced by 6 ± 12/5 ± 7 mmHg when compared with baseline (*P* = 0.02/<0.01). However, self-monitored blood pressure measurements data showed no decreases. MSNA did not change after RDN: 48 (31) bursts/min and 75 (23) bursts/100 heartbeats at 6-month follow-up when compared with 48 (41) bursts/min (*P* = 0.86) and 74 (48) bursts/100 heartbeats (*P* = 0.80) at baseline. In addition, no significant differences in HRV measures were observed 12 months after RDN. Their results showed that RDN did not change fasting glucose, median insulin sensitivity nor systemic sympathetic activity.^15^ The difference between this study and the previously published studies may be explained by patient selection, as the DREAMS-Study included nearly drug-naive patients whose MSNA was higher than other studies.^60,61^ As preclinical studies have proven that surgical denervation could lower sympathetic activity,^62^ another explanation may be that the currently used catheter did not adequately lower sympathetic nervous system activity. It is vital to realize that this study lacked a control group and T2DM sample capacity was small and made it difficult to draw firm conclusions. Therefore, further evaluation in rigorously designed clinical trials will be necessary to confirm previously reported benefits of RDN in patients with T2DM.

### Future Perspectives

In spite of the preliminary results observed by RDN in diabetic patients, several limitations have to be mentioned. Firstly, the current published studies lacked a proper control group, along with the sample capacity was small. Also, data obtained in the subgroups of diabetic patients were not separately analyzed and the follow-up period was very short. In addition, a reduction in blood pressure accounts for the improvements in glucose metabolism and insulin resistance cannot be excluded.

These limitations require future studies in the metabolic area to confirm the previous results. Appropriately designed future researches are needed to enhance the understanding of the potential diabetic consequences of RDN, as well as to document the durability of the results. Currently, ongoing clinical trials, including Renal Denervation in Diabetes (NCT 02081989, conducted to investigate the effects of RDN on diabetes), Metabolic Syndrome Study (Renal Sympathetic Denervation in Metabolic Syndrome, NCT 01911078, performed to determine the effects of RDN on insulin resistance and MSNA in patients with metabolic syndrome), and others, which are evaluating the effects of sympathetic RDN on glucose metabolism and insulin sensitivity, will further define the applicability of RDN in T2DM.

Besides reduction of blood pressure and improvements in glycemic control, reducing sympathetic activity by RDN might also have beneficial effects of reducing heart rate,^63^ improving atrial and ventricular arrhythmias^50^ and other currently unknown multiple effects. As long as the pathophysiological understanding of RDN therapy is confirmed by proper designed clinical trials, this technique could be used to treat a variety of diseases.

## CONCLUSION

In most studies, RDN achieved via novel catheter-based approach using radiofrequency energy has gained a significant role in resistant hypertension, as well as improvements in glycemic control in T2DM. This conforms the concept that activation of renal sympathetic nerves importantly contributes to insulin resistance and impairment of glucose metabolism. But the DREAMS-Study showed that RDN did not change median insulin sensitivity nor systemic sympathetic activity. Thus, more clinical trials are needed before any definite conclusion can be drawn. Unless the favorable result of better glucose metabolism is confirmed in large-scale, randomized studies, RDN may emerge as a novel therapeutic option for patients with T2DM.
